# Risk Assessment of Lead and Cadmium Exposure Through Raw Milk Consumption from Small-Scale Dairy Systems in the Central Peruvian Andes

**DOI:** 10.3390/toxics14050385

**Published:** 2026-04-30

**Authors:** Doris Chirinos-Peinado, Jorge Castro-Bedriñana, Elva Ríos-Ríos, Gianfranco Castro-Chirinos, Mery Baquerizo-Canchumanya

**Affiliations:** 1 Food and Nutritional Security Research Group, Universidad Nacional del Centro del Perú, Huancayo 12000, Peru; dchirinos@uncp.edu.pe; 2 Faculty of Sciences, Universidad Nacional Agraria La Molina, Lima 15012, Peru; erios@lamolina.edu.pe; 3 Faculty of Psychology, Universidad Peruana de Ciencias Aplicadas, Lima 15086, Peru; pcpsgcas@upc.edu.pe; 4 Faculty of Applied Sciences of Tarma, Universidad Nacional del Centro del Perú, Tarma 12651, Peru; mbaquerizo@uncp.edu.pe

**Keywords:** lead, cadmium, risk assessment, food safety, cow milk, Andean highlands, hazards models

## Abstract

Bovine milk is a primary dietary source of nutrients and bioactive compounds. However, its safety is increasingly under threat due to contamination from mining and intensive agriculture. In the Peruvian Andes, where small-scale dairy farming coexists with historical environmental liabilities, identifying the transfer of metals into the food chain is essential for public health. This study quantifies the concentrations of lead (Pb) and cadmium (Cd) in raw milk from small-scale producers in rural districts in the province of Huancayo. Non-carcinogenic risks for populations aged 2–85 years were assessed under three consumption scenarios. Forty-five samples were analyzed using microwave plasma atomic emission spectrometry (MP-AES). The mean concentrations of Pb and Cd were 11.30 ± 18.94 µg/kg and 7.85 ± 18.11 µg/kg, respectively, which are below the maximum permissible limits (MPL). However, spatial analysis identified critical hotspots near smelters, where Pb levels reached 103 µg/kg, which is a significant exceedance of the MPL of 20 µg/kg. Toxicological modelling showed that the Hazard Index (HI) remained below the unity threshold (HI < 1) for all scenarios, ruling out immediate systemic risks. Nevertheless, the highest HI (0.78) was observed in two-year-old children in the high-consumption scenario, highlighting a localized neurodevelopmental concern. These findings emphasize the importance of georeferenced environmental monitoring and differentiated public health policies to mitigate the chronic low-level exposure to metals in vulnerable, high-altitude populations.

## 1. Introduction

Bovine milk is a dietary staple recognized for its high nutritional density and immunomodulatory properties [1,2,3]. However, the presence of potentially toxic elements (PTEs) such as lead (Pb) and cadmium (Cd) poses a significant threat to its safety. These contaminants are a major public health concern globally, particularly in regions where agriculture overlaps with historical and ongoing mining and metallurgical activities [4,5]. Unlike essential minerals, Pb and Cd have no known biological function and act as potent systemic toxins [6]. Chronic exposure has been linked to irreversible neurodevelopmental impairment, nephrotoxicity, and endocrine disruption [4,7,8,9,10,11,12,13,14,15,16,17]. In particular, Pb interferes with neurotransmission and induces oxidative stress in the developing brain, leading to cognitive and behavioural deficits even at low exposure levels [10,18,19,20,21].

In high-altitude tropical ecosystems, the biogeochemical cycling of metals is influenced by unique environmental pressures. Atmospheric deposition from legacy smelting activities, the intensive use of phosphate fertilizers, and irrigation with mining-impacted water facilitate the transfer of PTEs from soil to forage and, ultimately, into the dairy food chain [22,23,24,25,26,27,28].

In Peru, where smallholder dairy systems produce most of the fresh milk consumed by local populations, surveillance is often fragmented or non-existent. The central highlands, located within the influence zone of the La Oroya Metallurgical Complex (LOMC), are a critical region in which to study the exposome of rural populations, which provides most of the dairy production [29].

The selection of Pb and cadmium Cd as the main focus of this study is justified by their high systemic toxicity and bioaccumulation capacity within the dairy food chain, as previously mentioned, as well as their frequent association with mining and metallurgical activities, which are prevalent in the central highlands of Peru [30,31,32]. It is important to consider them together in order to evaluate the risk posed by these potentially toxic metals in dairy systems that have historically been exposed to anthropogenic sources.

While previous studies have identified PTEs in isolated Andean hotspots [30,31,32,33,34], there is a lack of spatially resolved research integrating analytical chemistry with age-stratified human health risk modelling.

This study addresses this gap by evaluating Pb and Cd concentrations in raw milk produced by smallholder dairy farms with ≤5 cows) in 12 rural districts of Huancayo. We hypothesize that while average concentrations may comply with international standards, localized anthropogenic pressures create pockets of high exposure that significantly narrow safety margins for consumers aged 2–85 years. By combining robust MP-AES quantification with weekly intake (WI) and hazard index (HI) modelling, this work provides essential evidence for managing toxicological risks in mining-influenced agricultural landscapes.

## 2. Materials and Methods

### 2.1. Study Area and Environmental Context

The study was conducted across 12 districts in the province of Huancayo (3267–3902 m a.s.l.), which is characterized by a temperate highland climate and proximity (112–170 km) to the LOMC (Table 1).

**Figure 1:**
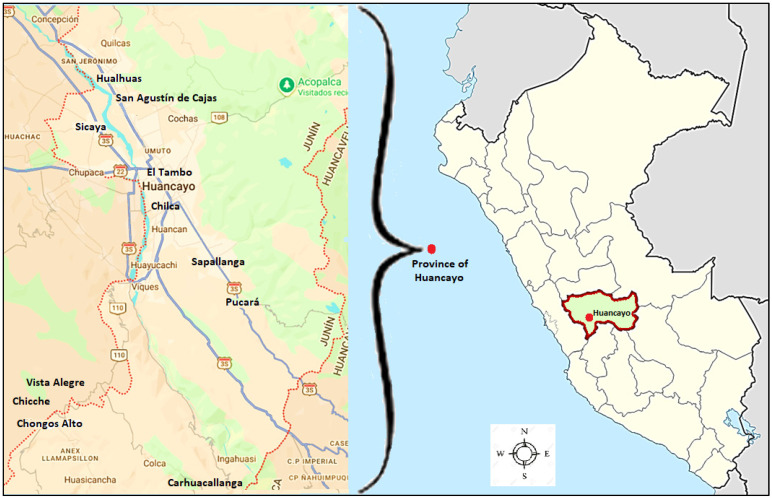
Map of the study area. 12 districts of the province of Huancayo 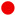. Source: Google Maps (https://www.google.com/maps/place/Huancayo/@-12.1803375,-75.2544988,27614m/data=!3m1!1e3!4m6!3m5!1s0x910e9cec4fc2eea1:0x82375e6f3f0e22a0!8m2!3d-11.9932468!4d-75.201229!16s%2Fm%2F02pv2dx?entry=ttu&g_ep=EgoyMDI2MDQyNi4wIKXMDSoASAFQAw%3D%3D, access date on 4 February 2026).

This region is a clear example of diffuse pollution. These inorganic pollutants are released into the water, soil, and atmosphere due to the rapid growth of agriculture and the metallurgical industry, inadequate waste management, and the use of fertilizers and pesticides [35]. Atmospheric particulate matter and current irrigation practices using water from the Mantaro River contribute to a complex matrix of heavy metal exposure. The geographic coordinates of the study area are approximately 12°04′05″ S and 75°12′38″ W (Figure 1).

This agroecological zone is characterized by small-scale agriculture, the use of phosphate fertilizers, and historical atmospheric deposition of metal-bearing particulates. Depending on the prevailing meteorological conditions, emissions from the LOMC may be transported over long distances and deposited in soils, potentially contributing to the accumulation of metals in pastures and food chains. These conditions make the region a relevant setting for evaluating heavy metal contamination in animal-derived food matrices.

### 2.2. Livestock Management and Feeding Practices

Dairy production in the study area is predominantly carried out by smallholder farmers using extensive, low-input systems. Herds typically consist of four to five cows, with milk yields ranging from three to five liters per cow per day. Animals are milked manually once a day, usually in the morning. Cattle are fed a diet of natural and cultivated pastures, including maize stubble (*Zea mays*), alfalfa (*Medicago sativa*), perennial ryegrass (*Lolium perenne*), and red clover (*Trifolium pratense*), supplemented with crop residues. No commercial concentrates are used. Reproduction occurs through natural mating, and technical assistance is limited. These relatively homogeneous management conditions minimize confounding variability related to feeding and husbandry.

### 2.3. Milk Sampling and Technical Implementation

A cross-sectional study was conducted in 2025. A total of 45 samples of raw milk (150 mL each) were collected directly from the morning milking of 45 individual cows on 24 small farms. The farms were selected based on herd size and stage of lactation. Collection took place under strict hygienic conditions.

To prevent potential contamination and preserve analyte integrity, all high-density polyethylene and glass containers were washed with 10% *w*/*w* nitric acid (HNO_3_) by weight and rinsed with deionized water prior to use.

The samples were stored at −4 °C and transported to the laboratory within 12 h in insulated containers with ice packs.

### 2.4. Instrumental Analysis and Quality Control

Prior to analysis, the samples were subjected to wet acid digestion in a hot block digestion system to destroy organic matter. This involved adding concentrated nitric acid (HNO_3_) and hydrogen peroxide (H_2_O_2_) at a controlled temperature until complete mineralization was achieved. Then, a 25 mL of milk was sampled and 1 mL each of HNO_3_ and H_2_O_2_ (in a 1:1 ratio) was added. The mixture was heated at 85 °C for two hours until the solution was clear and free of organic particles. Once cooled, the solution was filtered through a 0.45 µm membrane and transferred to a 25 mL volumetric flask. The solution was diluted to the mark with ultrapure water prior to instrumental analysis.

Quantification was performed using microwave plasma atomic emission spectroscopy (MP-AES; Agilent 4200, Agilent Technologies, Santa Clara, CA, USA) at the accredited laboratory of the National Institute of Agricultural Innovation (INIA-Huancayo), in accordance with the analytical procedures outlined in [36].

To ensure quality assurance, certified reference materials (CRMs) that are traceable to NIST and ISO 17034, this standard specifies the general quality assurance procedures for the production of all types of reference materials [37], and which contain 1000 mg/L of Pb and Cd (Inorganic Ventures, Christiansburg, VA, USA) were used to prepare the calibration curves and verify their accuracy. An analytical calibration curve was then obtained for each element, with a correlation coefficient (R^2^) of at least 0.995. Reagent blanks, analytical replicates and routine calibration performance verification were included.

The MP-AES operating conditions considered wavelengths of 220.353 nm for Pb and 226.502 nm for Cd. The limit of detection (LOD) for both metals was 0.001 mg/L (1 µg/kg), and the LOD was calculated as three times the standard deviation (σ) of ten consecutive readings from a digested milk sample.

Quality control and assurance procedures included reagent blank analysis, replicate analysis and routine calibration verification to ensure analytical stability, accuracy, precision, and reproducibility of the quantifications. Lead and cadmium concentrations in milk were reported in micrograms per kilogram (µg/kg), which is also the unit used for health risk assessment.

### 2.5. Exposure Modeling and Risk Characterization

Chronic dietary exposure was estimated by calculating the weekly intake (WI), which was derived from measured concentrations, with age-specific milk consumption rates and body weights for individuals aged 2–85 years, as obtained from national anthropometric data [38].

Three exposure scenarios (low, medium, and high milk consumption) were considered for each age group, based on international and regional consumption data [8,39,40,41]. The EWI values were then compared with the tolerable weekly intake (TWI) benchmarks established by the EFSA and the JECFA [42,43,44,45].

Daily milk intake in the low, medium, and high consumption scenarios was 400 d/day, 500 g/day and 600 g/day for children aged 2–5 years; 500 g/day, 600 g/day and 720 g/day for individuals aged 6 to 19 years; and 100 g/day to 250 g/day for individuals aged 20 to 85 years [46,47,48]. Using these values, a continuous baseline of dairy consumption was established for ages 2 to 85 years, enabling the estimation of WI by age and consumption scenario.

Non-carcinogenic systemic risk was characterized using the Target Hazard Quotient (THQ) and the Hazard Index (HI) [49,50]:

$$THQ=\frac{EF*ED*Wmilk*Cmetal}{RfD*Body\;weight*TA}$$

where

*EF* is the frequency of exposure (365 days a year).
*ED* is the exposure period equivalent to the average lifespan of an adult. For Peru, it is estimated at 76.5 years.
*Wmilk* is the daily consumption of milk (Kg/person/day).
*Cmetal* is the concentration of metal in milk (µg/kg).
*RfD* is the oral reference dose, which for the case of Cd and Pb used values of 1 and 3.5 µg/kg of body weight/day, respectively [51,52,53,54].
*Body weight*, kg for age.
*TA* is the average useful life, which is 27,922.5 days.

The exposure duration used in the THQ and HI calculations represents average lifetime exposure, in line with USEPA guidelines for assessing chronic non-cancer risk.

Age-specific variability is accounted for by considering body weight and daily milk consumption rates in different scenarios. This allows us to evaluate whether exposure at a young age could pose a significant long-term cumulative risk, even if exposure only occurs during a small part of a lifetime. The HI (Hazard Index) is the sum of the THQ values for Pb and Cd (HI = THQ Pb + THQ Cd). An HI threshold greater than 1 indicates a significant toxicological risk.

The model considered a continuous age range of 2–85 years, enabling specific vulnerability periods to be identified throughout the life cycle.

### 2.6. Statistical Analysis

The data were expressed as the mean ± standard deviation. Normality was assessed using the Shapiro–Wilk test. As the data did not follow a normal distribution, the differences in Pb and Cd concentrations among districts were evaluated using the Kruskal–Walli’s test, followed by Games–Howell post hoc comparisons. Statistical analyses were performed using IBM SPSS Statistics v23, with significance set at *p* < 0.05.

## 3. Results

### 3.1. Pb and Cd Concentrations in Raw Milk and Comparison with International Limits

MP-AES analysis revealed that, although there is extreme variability, the average heavy metal concentrations in the Huancayo Province generally remain within international safety standards, albeit with extreme variability. The mean concentration of Pb was 11.30 ± 18.94 µg/kg, while Cd averaged 7.85 ± 18.11 µg/kg (Table 2). However, it is important to note that, while the overall means comply with the MPL, 13.33% of Pb samples and 8.89% of Cd samples exceeded the safety thresholds of 20 µg/kg and 10 µg/kg, respectively [55,56].

The detection of peak concentrations of 103 µg/kg for Pb and 75 µg/kg for Cd in certain districts highlights a severe localized contamination issue, representing more than five and seven times the legal threshold, highlighting a severe localized contamination issue, respectively. This level of non-compliance is of particular concern for small-scale dairy systems in the central Peruvian Andes, where raw milk is often distributed directly to local markets without prior industrial processing or dilution. This could potentially expose consumers to acute and chronic metal concentrations. The high coefficients of variation (167.26% for Pb and 229.11% for Cd) confirm the presence of localized “hotspots” of contamination within the region.

### 3.2. Spatial Variability and Identification of Hotspots

The spatial distribution of these exceedances was not random; rather, they were concentrated in districts subject to higher levels of anthropogenic pressure. The high percentage of samples exceeding the MPL for Pb (13.33%) suggests widespread environmental dispersion, likely through atmospheric deposition from mining activities or the use of contaminated irrigation water in the Mantaro Valley. The exceedance rate of 8.89% for Cd is closely linked to specific districts such as El Tambo, where mean concentration (33.58 mg/kg) was more than three times the MPL. This suggests a strong correlation with phosphate fertilizer runoff or industrial waste lixiviates (Table 3).

Analysis by district revealed statistically significant differences (*p* < 0.05). El Tambo district had the highest concentrations of both metals, exceeding the MPL in both cases. In contrast, districts such as Chicche and Vista Alegre showed minimal levels, close to the detection limit. Other districts such as Chilca (15.0 µg/kg Pb) and Sicaya (16.2 µg/kg Pb) showed levels close to the critical limit, but without exceeding it on average.

### 3.3. Dietary Exposure Assessment (Weekly Intake)

**Figure 2:**
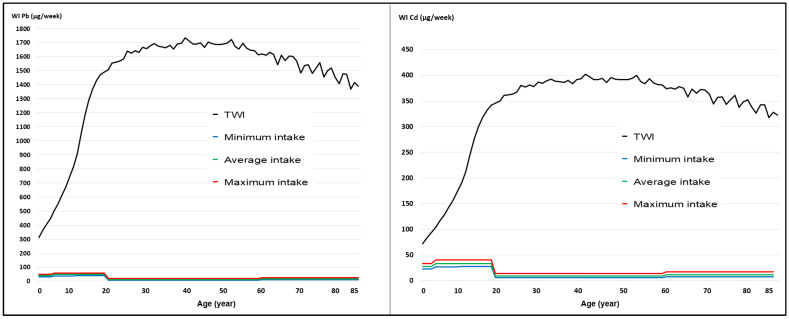
Weekly intake (WI) for Pb and Cd in three milk consumption scenarios.

The modelled weekly intake (WI) curves for Pb and Cd show that, even in the maximum consumption scenario, levels remain significantly below the tolerable weekly intake (TWI) established by international bodies. A pronounced increase in cumulative intake is observed between ages 2 and 20, stabilizing during adulthood and declining slightly after the age of 65. This reflects the typical milk consumption patterns of the Peruvian population (Figure 2).

Under low, medium, and high milk consumption scenarios, the average TWI for Pb in the Peruvian population aged 2–85 years was 14.83, 20.40, and 27.75 µg/kg, respectively, while for Cd it was 10.37, 14.26, and 19.40 µg/kg. The average TWIs for Pb and Cd were 1447 and 336 µg/kg, respectively, across the three milk consumption scenarios.

### 3.4. Health Risk Characterization (THQ and HI)

Although weekly intake remains low, the analysis of the Target Hazard Quotient (THQ) and Hazard Index (HI) reveals significant age-related vulnerability. In all cases evaluated, however, the HI was less than 1.0 (Table 4).

**Figure 3:**
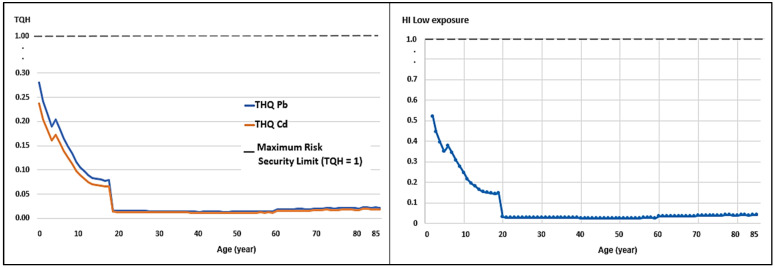
Target Hazard Coefficient (THQ) for Pb and Cd and Risk Index (HI) for low milk consumption. An HI threshold greater than 1 indicates a significant toxicological risk.

**Figure 4:**
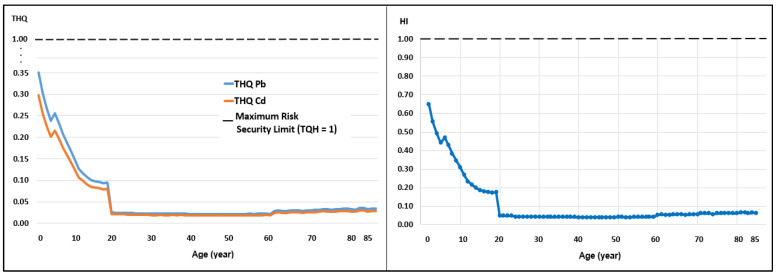
Target Hazard Coefficient (THQ) for Pb and Cd and Risk Index (HI) for medium milk consumption. An HI threshold greater than 1 indicates a significant toxicological risk.

**Figure 5:**
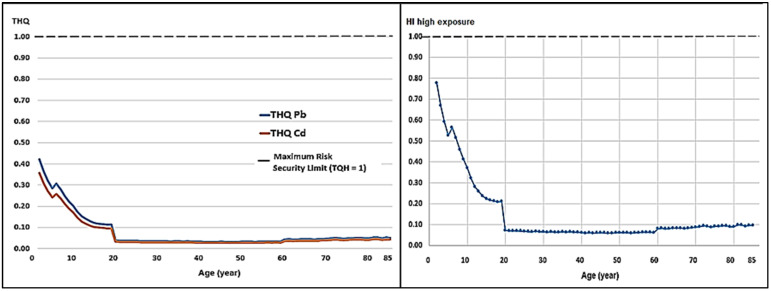
Target Hazard Coefficient (THQ) for Pb and Cd and Risk Index (HI) for high milk consumption. An HI threshold greater than 1 indicates a significant toxicological risk.

Respecting the impact on different age groups, the risk is inversely proportional to age. Children aged two presented the highest HI values across all scenarios: 0.52 (low), 0.65 (medium), and 0.78 (high) (Table 5; Figure 3, Figure 4 and Figure 5). By contrast, for adults aged 25, the HI under a high consumption scenario was only 0.07. This implies that a toddler faces a systemic risk that is 11 times higher than that faced by an adult consuming the same milk.

With regard to consumption scenarios, the analysis of variance revealed significant differences (*p* < 0.05) in exposure levels. The high exposure scenario increased the combined systemic risk (HI) to a mean of 0.14 for the general population, compared to 0.08 for the low exposure scenario (Table 5).

The lead and cadmium contents in milk from small production units in the province of Huancayo, the THQ and HI values in the low, medium, and high exposure scenarios are shown as Supplementary Materials in Tables S1–S4.

## 4. Discussion

### 4.1. Environmental Dynamics of Localized Pollution

As shown in Table 3, the finding that 13.33% of the samples exceeded the maximum permissible limit (MPL) for Pb and 8.89% for Cd highlights a significant safety gap in small-scale Andean dairy systems. In the central Peruvian highlands, the proximity of pastures to the Mantaro River and historical smelting centers such as La Oroya facilitates the transfer of metals through multiple pathways.

Previous studies in other mining-impacted regions of South America have shown that Pb-rich particulates can be deposited in the atmosphere and settle on soil and vegetables, including English ryegrass (*Lolium perenne*) and Kikuyu grass (*Pennisetum clandestinum*) [57,58,59], the primary forage in Huancayo [60]. Furthermore, the maximum Pb value detected in raw milk (103 µg/kg) is likely the result of cattle ingesting contaminated forage or drinking water from irrigation channels impacted by mining tailings, a common issue in the Peruvian highlands [61,62,63].

Exceeding the LMPs in specific districts such as El Tambo suggests that contamination is not a uniform regional phenomenon, but rather responds to localized anthropogenic pressures [64].

The observed spatial heterogeneity, particularly in districts such as El Tambo, is consistent with international studies demonstrating that contamination by potentially toxic metals in dairy products is strongly modulated by local anthropogenic sources, vehicular traffic, and small-scale industries [15,65,66,67,68,69,70].

A similar study analyzed the relationship between lead (Pb) and cadmium (Cd) concentrations and the physical and chemical composition of milk. It reported average Pb and Cd concentrations that were slightly lower than those in our study because it did not include farms located near mineral processing plants, unlike our study. The study reported weak negative correlations between Pb/Cd and protein and lactose, as well as moderate correlations with pH [71].

Compared to other countries, the results are lower than those reported in India, Indonesia, and Egypt [9,65], but similar to organic or rural systems in Poland, Ethiopia, and Turkey [70]. This reflects the direct influence of the level of industrialization and agronomic practices on contamination on the potentially toxic metals.

Taken together, the findings highlight the importance of strengthening toxicological surveillance systems and geographic monitoring programmes, and of implementing mitigation measures in areas near mining and industrial sites, to ensure the safety of milk produced by small-scale farmers. This is crucial to protecting the health of population that depend on milk as a primary source of nutrients.

Proximity to mineral processing plants and solid waste deposits in these areas facilitates the transfer of Pb and Cd to the soil and pastures, ultimately integrating them into the dairy chain. Smelting emissions not only increase the concentrations of Cd and Pb in the topsoil but also their mobility and bioavailability [64]. These “hot spots” of up to 103 µg/kg of Pb demonstrate that monitoring based on district averages can mask severe risks at the farm level.

### 4.2. Neurotoxicological Implications and Child Vulnerability

The results of the Target Hazard Quotient (THQ) and the Hazard Index (HI) are particularly concerning for the pediatric population (Table 5). Although the HI remains below 1.0, the value of 0.78 in two-year-olds (Figure 5) suggests that milk consumption alone accounts for almost 80% of the safe daily intake margin for these toxins.

The greater vulnerability observed in the 2- to 5-year-old group in this study can be explained by physiological and behavioral factors specific to this developmental stage. Children in this age group have a higher rate of food and water intake per unit of body weight and absorb metals such as Pb and Cd more efficiently in the gastrointestinal tract. They also have lower renal excretion rates. Furthermore, their detoxification systems, including liver and kidney function, are not yet fully developed. This restricts their excretion capacity and encourages bioaccumulation [72].

Behaviorally, the diet at this stage tends to rely more heavily on staple foods such as milk, thereby increasing exposure. Taken together, these characteristics would increase susceptibility to toxic effects, particularly in developing organs such as the nervous system. This reinforces the need to prioritize this age group in surveillance and risk management strategies.

The scientific literature maintains that there is no safe threshold for lead exposure, particularly during critical stages of brain development [73,74,75]. Pb^2+^ acts as a calcium (Ca^2+^) mimetic, interfering with neurotransmitter release and synaptic plasticity and leading to irreversible cognitive deficits [19,76,77]. Regarding Cd, the 8.89% exceedance rate (Table 3) poses a long-term metabolic risk. Cd is a known metalloestrogen and nephrotoxin, with a biological half-life exceeding 20 years. Exposure in early childhood through milk can cause chronic bioaccumulation in the renal cortex, potentially leading to Fanconi syndrome or bone demineralization in adulthood [78].

This is of particular concern in the Andean context, where dietary and environmental factors can exacerbate metal absorption. The Pb-Cd synergy detected in El Tambo due to co-exposure would increase cellular oxidative stress. While Pb affects cognitive development, Cd exerts widespread toxic effects on human health through various biochemical and molecular mechanisms, initiating a process of renal bioaccumulation that will last for decades, given its biological half-life of up to 30 years [79,80].

### 4.3. Scenario Assessment and Food Safety

The stability of WI curves below the TWI indicates that milk is generally safe for widespread consumption. However, the safety margin narrows dramatically during the first five years of life due to the higher rate of milk intake per kilogram of body weight [81]. This study demonstrates that, while essential for local food security, smallholder systems require differentiated monitoring to prevent milk from becoming a source of chronic exposure to heavy metals in areas influenced by mining activities.

While this study focused on evaluating lead (Pb) and cadmium (Cd) as priority trace metals, it is important to recognize that in contexts influenced by mining and metallurgical activities, the presence of other potentially toxic elements such as arsenic (As), mercury (Hg), chromium (Cr), aluminium (Al) and nickel (Ni) is highly likely. The literature has demonstrated that exposure to multiple metals simultaneously intensifies their toxic effects through synergistic interactions, thereby increasing the toxicological risk beyond that estimated for individual compounds. Moderate positive relationships have been found among these toxic compounds [82]. While the THQ and HI values for Pb and Cd suggest limited non-carcinogenic risk at the population level, the total exposure burden could be underestimated by failing to consider the combined contribution of other contaminants present in the environment. This is particularly relevant in vulnerable populations, such as children, where cumulative exposure to mixtures of metals can amplify adverse effects on neurological and metabolic development. Therefore, future studies should adopt a multi-element approach to allow for a more comprehensive risk assessment and a better understanding of real-world exposure scenarios in complex agro-environmental systems.

### 4.4. Implications for the Smallholder Dairy Sector

Our results demonstrate a toxicological paradox, while the regional average suggests safety, the high variability (CV > 160%) means that one in every eight children consuming milk in districts such as El Tambo is likely to be exposed to Pb levels exceeding international safety limits (Table 3 and Table 4). Unlike large industrial dairy processors who use bulking to dilute contaminants by mixing milk from many farms, smallholders in the Andes often sell milk directly to local consumers. This lack of a dilution makes the 13.33% non-compliance rate a direct public health threat as the provisional tolerable weekly intake is no longer appropriate given the lack of evidence for a threshold for critical lead-induced effects [83]. Protecting children from the potential risk of neurodevelopmental effects would also protect them against all other adverse effects of lead across all populations.

### 4.5. Limitations, Strengths and Future Perspectives

Despite the methodological challenges it faced, this study has made significant scientific contributions in the Andean context. The limitations include the observational and cross-sectional design, which prevents the establishment of mechanical causality, and the exploratory district-level sample size, which should be interpreted with caution when making geographical comparisons. Furthermore, the national average body weights used in the study may not accurately represent the local highland population.

However, the strengths of this work are substantial. This pioneering study provides an integrated, multi-spatial assessment of lead and cadmium risk among small-scale producers in the Central Andes, where the La Oroya Metallurgical Complex has operated since 1922. Analytical rigor and risk modelling by age group (2–85 years) have allowed early childhood to be identified as the most vulnerable period, transforming descriptive data into public health indicators. Looking ahead, this work lays the groundwork for future longitudinal research and expanded sampling designs. The study’s main contribution is providing evidence to inform the development of food surveillance policies and protect rural populations in environmentally vulnerable high Andean ecosystems.

## 5. Conclusions

This study shows that although milk produced by small-scale farmers in Peru’s central highlands generally meets international standards, critical contamination points validate the influence of human activities in the dairy supply chain. Peak levels of lead (Pb) and cadmium (Cd) levels of up to 103 µg/kg and 75 µg/kg, respectively, have been found in districts such as El Tambo. The extreme geospatial variability (CV > 160%) confirms that the risk depends on specific emission sources. While the Hazard Index (HI) remained below 1 for the general population, children aged 2–5 are critically vulnerable, with an HI of 0.78 in high-consumption scenarios. This indicates a narrow safety margin for neurotoxins, for which there is no safe exposure threshold.

Consequently, integrating MP-AES quantification with multiscenario modelling is established as a vital diagnostic tool for epidemiological surveillance in mining regions. Continuous georeferenced monitoring of milk, soil and water is recommended, particularly focusing on blood lead screening protocols for early childhood. Similarly, enhancing environmental oversight of local industries and educating producers in risk management is crucial to mitigating the bioaccumulation of metals. This will lay the groundwork for future longitudinal research into the chronic metabolic effects on high-altitude Andean populations.

## Figures and Tables

**Table 1:** Altitude and characteristics of the districts included in the study.

District	Average Altitude, m.a.s.l	n	Geographic Characteristic
Hualhuas	3267	2	Area: 14 km^2^. Geographic coordinates: Latitude: −11.9714, Longitude: −75.2508, Latitude: 11°58′17″ South, Longitude: 75°15′3″ West
Chilca	3273	5	Area: 29 km^2^. Geographic coordinates: Latitude: −12.0867, Longitude: −75.2083, Latitude: 12°5′12″ South, Longitude: 75°12′30″ West
Vista alegre (Population Center)	3276	2	Area: 1.8 km^2^. Geographic coordinates: Latitude: −12.0804, Longitude: −75.2880, Latitude: 12°4′49″ South, Longitude: 75°17′17″ West
Sapallanga	3285	4	Area: 119 km^2^. Geographic coordinates: Latitude: −12.148424, Longitude: −75.158758, Latitude: 12°08′54″ South, Longitude: 75°09′32″ West
San Agustín de Cajas	3297	2	Area: 26 km^2^. Geographic coordinates: Latitude: −11.9897, Longitude: −75.2442, Latitude: 11°59′23″ South, Longitude: 75°14′39″ West
El Tambo	3305	8	Area: 167 km^2^. Geographic coordinates: Latitude: −12.0503, Longitude: −75.2214, Latitude: 12°3′1″ South, Longitude: 75°13′17″ West
Sicaya	3305	5	Area: 41 km^2^. Geographic coordinates: Latitude: −12.0147, Longitude: −75.28, Latitude: 12°0′53″ South, Longitude: 75°16′48″ West
Pucará	3374	3	Area: 110.5 km^2^. Geographic coordinates: Latitude: −12.1726, Longitude: −75.14547, Latitude: 12°10′20″ South, Longitude 75°08′50″ West
Chongos Alto	3573	4	Area: 41 km^2^. Geographic coordinates: Latitude: −12.3117, Longitude: −75.2892, Latitude: 12°18′42″ South, Longitude: 75°17′21″ West
Chicche	3606	3	Area: 42 km^2^. Geographic coordinates: Latitude: −12.2961, Longitude: −75.2986, Latitude: 12°17′46″ South, Longitude: 75°17′55″ West
Carhuacallanga	3774	2	Area: 13 km^2^. Geographic coordinates: Latitude: −12.355, Longitude: −75.2006, Latitude: 12°21′18″ South, Longitude: 75°12′2″ West
Acopalca (Peasant Community)	3902	5	Area: 378.4 km^2^. Geographic coordinates: Latitude −11.9876, Longitude: −75.10193, Latitude 11°59′15″ south, Longitude 75°6′7″ west

n: number of samples collected by district, based on the presence of cows in production in family farms. m.a.s.l: meters above sea level.

**Table 2:** Lead and cadmium in milk concentration and comparison with the LMP for whole milk (µg/kg).

Variable	Mean	SD	CV, %	Min	Max	MPL, µg/kg
Pb, µg/kg	11.30	18.94	167.26	0.90	103.0	20.0
Cd, µg/kg	7.85	18.11	229.11	0.90	75.0	10.0

SD: Standard deviation; CV: Coefficient of variation; Min: Minimum; Max: Maximum; MPL: Maximum Permissible Limit.

**Table 3:** Concentration of Pb and Cd in whole cow’s milk, produced on farms of small producers in the central highlands of Peru (µg/kg).

District	Pb				Cd			
	Min	Max	Mean	SD	Min	Max	Mean	SD
El Tambo	4.00	103.00	30.63 a	38.29	0.90	75.00	33.58 a	33.35
Chilca	8.00	21.00	15.00 b	4.85	0.90	9.00	5.98 ab	3.43
Sicaya	3.00	27.00	16.20 b	9.01	0.90	9.00	2.54 ab	3.61
Hualhuas	9.00	11.00	10.00 c	1.41	2.00	3.00	2.50 ab	0.71
San Agustín de Cajas	6.00	13.00	9.50 c	4.95	3.00	3.00	3.00 ab	0.00
Chongos Alto	1.00	17.00	5.57 c	7.54	0.90	9.00	2.93 ab	4.05
Pucará	3.00	7.00	4.67 c	2.10	0.90	2.00	1.30 ab	0.61
Sapallanga	1.00	9.00	4.00 c	3.56	0.90	2.00	1.20 ab	0.55
Carhuacallanga	2.00	3.00	2.50 c	0.71	0.90	1.00	0.95 b	0.70
Acopalca	0.90	2.00	1.16 c	0.47	0.90	0.90	0.90 b	0.00
Vista Alegre	0.90	1.00	0.95 c	0.10	0.90	0.90	0.90 b	0.00
Chicche	0.90	0.90	0.90 c	0.00	0.90	0.90	0.90 b	0.00

a, b, c, Average values per metal for district, with different letters vary significantly (*p* < 0.05). Games-Howell non-parametric post hoc test.

**Table 4:** Summary of THQ and HI values in children and adults by exposure level.

Exposure	Age (Years)	THQ-Pb	THQ-Cd	HI
Low	2	0.28	0.24	0.52
	5	0.19	0.16	0.35
	25	0.02	0.01	0.03
Medium	2	0.35	0.30	0.65
	5	0.24	0.20	0.44
	25	0.02	0.02	0.04
High	2	0.42	0.36	0.78
	5	0.29	0.24	0.53
	25	0.04	0.03	0.07

**Table 5:** Target Hazard Coefficient (THQ) by level of exposure to Pb and Cd and Risk Index (HI).

Variable	Mean	Standard Deviation	Confidence Interval, 95%	
			Lower	Upper
THQ for Pb:				
Low exposure	0.044 b	0.06	0.031	0.057
Average exposure	0.058 ab	0.07	0.043	0.074
High exposure	0.077 a	0.08	0.058	0.095
THQ for Cd:				
Low exposure	0.037 b	0.05	0.026	0.048
Average exposure	0.049 ab	0.06	0.036	0.063
High exposure	0.065 a	0.07	0.049	0.080
HI:				
Low exposure	0.081 b	0.11	0.057	0.105
Average exposure	0.108 ab	0.13	0.079	0.137
High exposure	0.141 a	0.16	0.108	0.175

a, b, Different letters for each variable’s exposure level show significant differences (*p* < 0.05).

## Data Availability

The original contributions presented in this study are included in the article/Supplementary Materials. For any further inquiries, please contact the corresponding author.

## Supplementary Materials

The following supporting information can be downloaded at: https://www.mdpi.com/article/10.3390/toxics14050385/s1, Table S1: Lead and cadmium content in milk from small production units in the province of Huancayo (µg/kg); Table S2: Determination of THQ and HI in a low exposure scenario; Table S3: Determination of THQ and HI in a medium exposure scenario; Table S4: Determination of THQ and HI in a high exposure scenario.

## Abbreviations

The following abbreviations are used in this manuscript:

Pb	Lead
Cd	Cadmium
MP-AES	Microwave Plasma-Atomic Emission Spectrometry
MPL	Maximum Permissible Limits
THQ	Target Hazard Quotient
HI	Hazard Index
PTE	Potentially Toxic Elements
LOMC	La Oroya Metallurgical Complex
WI	Weekly Intake
TWI	Tolerable Weekly Intake
INIA	Instituto Nacional de Investigación Agraria
LOD	Limits of Detection
