# Supplementary material for: Risk Assessment of Lead and Cadmium Exposure Through Raw Milk Consumption from Small-Scale Dairy Systems in the Central Peruvian Andes
Source: Toxics. 2026 Apr 30;14(5):385. doi: 10.3390/toxics14050385 (PMC13211767; doi:10.3390/toxics14050385)
Supplement: Supplementary file 1 [file toxics-14-00385-s001.zip › toxics-4231508-SI.pdf]

Supplementary material

Table S1. Lead and cadmium content in milk from small production units in the province of Huancayo (µg/kg).

Location	Cd	Pb
Sapallanga	2.00	4.00
Sapallanga	0.90	2.00
Sapallanga	0.90	1.00
Sapallanga	0.90	9.00
Pucará	1.00	7.00
Pucará	2.00	4.00
Pucará	0.90	3.00
Chilca	0.90	17.00
Chilca	8.00	21.00
Chilca	9.00	16.00
Chilca	8.00	13.00
Chilca	4.00	8.00
Sicaya	0.90	13.00
Sicaya	1.00	3.00
Sicaya	0.90	21.00
Sicaya	0.90	27.00
Sicaya	9.00	17.00
Chongos Alto	0.90	2.00
Chongos Alto	0.90	1.00
Chongos Alto	0.90	3.00
Chongos Alto	9.00	17.00
Carhucallanga	1.00	3.00
Carhucallanga	0.90	2.00
Vista Alegre	0.90	1.00
Vista Alegre	0.90	0.90
Chicche	0.90	0.90
Chicche	0.90	0.90
Chicche	0.90	0.90
Acopalca	0.90	0.90
Acopalca	0.90	2.00
Acopalca	0.90	1.00
Acopalca	0.90	0.90
Acopalca	0.90	1.00
Hualhuas	2.00	11.00
Hualhuas	3.00	9.00
San Agustín de Cajas	3.00	6.00
San Agustín de Cajas	3.00	13.00
El Tambo	58.00	17.00
El Tambo	56.00	15.00
El Tambo	75.00	8.00
El Tambo	68.00	9.00
El Tambo	0.90	4.00
El Tambo	6.80	9.00
El Tambo	3.00	103.00
El Tambo	0.90	80.00

Table S2. Determination of THQ and HI in a low exposure scenario

Age (year)	Weight, kg	Contaminant occurrence in milk		Milk consumption	Reference TWI		Calculated		THQ		HI
		Pb (ug/kg)	Cd (ug/kg)	Kg/day	TWI Pb	TWI Cd	TWI Pb	TWI Cd	Pb	Cd	
2	12.40	11.30	7.90	0.400	310	72	310.00	71.92	0.28	0.24	0.52
3	14.40	11.30	7.90	0.400	360	84	360.00	83.52	0.24	0.20	0.45
4	16.10	11.30	7.90	0.400	403	93	402.50	93.38	0.21	0.18	0.39
5	17.90	11.30	7.90	0.400	448	104	447.50	103.82	0.19	0.16	0.35
6	20.10	11.30	7.90	0.480	503	117	502.50	116.58	0.20	0.17	0.38
7	22.00	11.30	7.90	0.480	550	128	550.00	127.60	0.19	0.16	0.34
8	24.60	11.30	7.90	0.480	615	143	615.00	142.68	0.17	0.14	0.31
9	26.80	11.30	7.90	0.480	670	155	670.00	155.44	0.15	0.13	0.27
10	29.60	11.30	7.90	0.480	740	172	740.00	171.68	0.13	0.11	0.25
11	32.70	11.30	7.90	0.480	818	190	817.50	189.66	0.12	0.10	0.21
12	36.40	11.30	7.90	0.500	910	211	910.00	211.12	0.11	0.09	0.19
13	41.90	11.30	7.90	0.500	1048	243	1047.50	243.02	0.10	0.08	0.18
14	47.60	11.30	7.90	0.500	1190	276	1190.00	276.08	0.09	0.08	0.16
15	51.70	11.30	7.90	0.500	1293	300	1292.50	299.86	0.08	0.07	0.16
16	54.70	11.30	7.90	0.500	1368	317	1367.50	317.26	0.08	0.07	0.15
17	57.30	11.30	7.90	0.500	1433	332	1432.50	332.34	0.08	0.07	0.15
18	58.90	11.30	7.90	0.500	1473	342	1472.50	341.62	0.08	0.07	0.14
19	59.60	11.30	7.90	0.500	1490	346	1490.00	345.68	0.08	0.07	0.15
20	60.30	11.30	7.90	0.096	1508	350	1507.50	349.74	0.02	0.01	0.03
21	62.20	11.30	7.90	0.096	1555	361	1555.00	360.76	0.02	0.01	0.03
22	62.40	11.30	7.90	0.096	1560	362	1560.00	361.92	0.02	0.01	0.03
23	62.70	11.30	7.90	0.096	1568	364	1567.50	363.66	0.02	0.01	0.03
24	63.30	11.30	7.90	0.096	1583	367	1582.50	367.14	0.02	0.01	0.03
**25**	**65.60**	11.30	7.90	0.096	1640	380	1640.00	380.48	0.02	0.01	0.03
26	65.00	11.30	7.90	0.096	1625	377	1625.00	377.00	0.02	0.01	0.03
27	65.70	11.30	7.90	0.096	1643	381	1642.50	381.06	0.02	0.01	0.03
28	65.20	11.30	7.90	0.096	1630	378	1630.00	378.16	0.02	0.01	0.03
29	66.70	11.30	7.90	0.096	1668	387	1667.50	386.86	0.01	0.01	0.03
30	66.30	11.30	7.90	0.096	1658	385	1657.50	384.54	0.01	0.01	0.03
31	67.10	11.30	7.90	0.096	1678	389	1677.50	389.18	0.01	0.01	0.03
32	67.70	11.30	7.90	0.096	1693	393	1692.50	392.66	0.01	0.01	0.03
33	67.00	11.30	7.90	0.096	1675	389	1675.00	388.60	0.01	0.01	0.03
34	66.80	11.30	7.90	0.096	1670	387	1670.00	387.44	0.01	0.01	0.03
35	66.50	11.30	7.90	0.096	1663	386	1662.50	385.70	0.01	0.01	0.03
36	67.20	11.30	7.90	0.096	1680	390	1680.00	389.76	0.01	0.01	0.03
37	66.20	11.30	7.90	0.096	1655	384	1655.00	383.96	0.01	0.01	0.03
38	67.60	11.30	7.90	0.096	1690	392	1690.00	392.08	0.01	0.01	0.03
39	67.80	11.30	7.90	0.096	1695	393	1695.00	393.24	0.01	0.01	0.03
40	69.30	11.30	7.90	0.094	1733	402	1732.50	401.94	0.01	0.01	0.03
41	68.50	11.30	7.90	0.094	1713	397	1712.50	397.30	0.01	0.01	0.03
42	67.60	11.30	7.90	0.094	1690	392	1690.00	392.08	0.01	0.01	0.03
43	67.60	11.30	7.90	0.094	1690	392	1690.00	392.08	0.01	0.01	0.03
44	68.00	11.30	7.90	0.094	1700	394	1700.00	394.40	0.01	0.01	0.03
45	66.60	11.30	7.90	0.094	1665	386	1665.00	386.28	0.01	0.01	0.03

46	68.20	11.30	7.90	0.094	1705	396	1705.00	395.56	0.01	0.01	0.03
47	67.70	11.30	7.90	0.094	1693	393	1692.50	392.66	0.01	0.01	0.02
48	67.50	11.30	7.90	0.094	1688	392	1687.50	391.50	0.01	0.01	0.02
49	67.50	11.30	7.90	0.094	1688	392	1687.50	391.50	0.01	0.01	0.03
50	67.60	11.30	7.90	0.094	1690	392	1690.00	392.08	0.01	0.01	0.03
51	67.90	11.30	7.90	0.094	1698	394	1697.50	393.82	0.01	0.01	0.03
52	68.90	11.30	7.90	0.094	1723	400	1722.50	399.62	0.01	0.01	0.03
53	66.90	11.30	7.90	0.094	1673	388	1672.50	388.02	0.01	0.01	0.03
54	66.20	11.30	7.90	0.094	1655	384	1655.00	383.96	0.01	0.01	0.03
55	67.80	11.30	7.90	0.094	1695	393	1695.00	393.24	0.01	0.01	0.03
56	66.40	11.30	7.90	0.094	1660	385	1660.00	385.12	0.01	0.01	0.03
57	65.80	11.30	7.90	0.094	1645	382	1645.00	381.64	0.01	0.01	0.03
58	65.70	11.30	7.90	0.094	1643	381	1642.50	381.06	0.01	0.01	0.03
59	64.50	11.30	7.90	0.094	1613	374	1612.50	374.10	0.01	0.01	0.03
60	64.80	11.30	7.90	0.120	1620	376	1620.00	375.84	0.02	0.02	0.03
61	64.40	11.30	7.90	0.120	1610	374	1610.00	373.52	0.02	0.02	0.04
62	65.20	11.30	7.90	0.120	1630	378	1630.00	378.16	0.02	0.02	0.03
63	64.60	11.30	7.90	0.120	1615	375	1615.00	374.68	0.02	0.02	0.03
64	61.70	11.30	7.90	0.120	1543	358	1542.50	357.86	0.02	0.02	0.04
65	64.40	11.30	7.90	0.120	1610	374	1610.00	373.52	0.02	0.02	0.04
66	62.90	11.30	7.90	0.120	1573	365	1572.50	364.82	0.02	0.02	0.04
67	64.20	11.30	7.90	0.120	1605	372	1605.00	372.36	0.02	0.02	0.03
68	64.00	11.30	7.90	0.120	1600	371	1600.00	371.20	0.02	0.02	0.04
69	62.70	11.30	7.90	0.120	1568	364	1567.50	363.66	0.02	0.02	0.04
70	59.30	11.30	7.90	0.120	1483	344	1482.50	343.94	0.02	0.02	0.04
71	61.50	11.30	7.90	0.120	1538	357	1537.50	356.70	0.02	0.02	0.04
72	61.70	11.30	7.90	0.120	1543	358	1542.50	357.86	0.02	0.02	0.04
73	59.20	11.30	7.90	0.120	1480	343	1480.00	343.36	0.02	0.02	0.04
74	60.70	11.30	7.90	0.120	1518	352	1517.50	352.06	0.02	0.02	0.04
75	62.30	11.30	7.90	0.120	1558	361	1557.50	361.34	0.02	0.02	0.04
76	58.20	11.30	7.90	0.120	1455	338	1455.00	337.56	0.02	0.02	0.04
77	60.10	11.30	7.90	0.120	1503	349	1502.50	348.58	0.02	0.02	0.04
78	60.70	11.30	7.90	0.120	1518	352	1517.50	352.06	0.02	0.02	0.04
79	58.20	11.30	7.90	0.120	1455	338	1455.00	337.56	0.02	0.02	0.04
80	56.30	11.30	7.90	0.120	1408	327	1407.50	326.54	0.02	0.02	0.04
81	59.10	11.30	7.90	0.120	1478	343	1477.50	342.78	0.02	0.02	0.04
82	59.00	11.30	7.90	0.120	1475	342	1475.00	342.20	0.02	0.02	0.04
83	54.70	11.30	7.90	0.120	1368	317	1367.50	317.26	0.02	0.02	0.04
84	56.60	11.30	7.90	0.120	1415	328	1415.00	328.28	0.02	0.02	0.04
85	55.60	11.30	7.90	0.120	1390	322	1390.00	322.48	0.02	0.02	0.04

Table S3. Determination of THQ and HI in a medium exposure scenario

Age (year)	Weight (kg)	Contaminant occurrence in milk		Milk consumption, kg/day	Reference TWI		Calculated		THQ		HI
		Pb (ug/kg)	Cd (ug/kg)		TWI Pb	TWI Cd	TWI Pb	TWI Cd	Pb	Cd	
2	12.40	11.30	7.90	0.500	310	72	310.00	71.92	0.35	0.30	0.65
3	14.40	11.30	7.90	0.500	360	84	360.00	83.52	0.30	0.26	0.56
4	16.10	11.30	7.90	0.500	403	93	402.50	93.38	0.27	0.23	0.49
5	17.90	11.30	7.90	0.500	448	104	447.50	103.82	0.24	0.20	0.44
6	20.10	11.30	7.90	0.600	503	117	502.50	116.58	0.25	0.22	0.47
7	22.00	11.30	7.90	0.600	550	128	550.00	127.60	0.23	0.20	0.43
8	24.60	11.30	7.90	0.600	615	143	615.00	142.68	0.21	0.18	0.38
9	26.80	11.30	7.90	0.600	670	155	670.00	155.44	0.19	0.16	0.34
10	29.60	11.30	7.90	0.600	740	172	740.00	171.68	0.17	0.14	0.31
11	32.70	11.30	7.90	0.600	818	190	817.50	189.66	0.14	0.12	0.27
12	36.40	11.30	7.90	0.600	910	211	910.00	211.12	0.13	0.11	0.23
13	41.90	11.30	7.90	0.600	1048	243	1047.50	243.02	0.12	0.10	0.21
14	47.60	11.30	7.90	0.600	1190	276	1190.00	276.08	0.11	0.09	0.20
15	51.70	11.30	7.90	0.600	1293	300	1292.50	299.86	0.10	0.09	0.19
16	54.70	11.30	7.90	0.600	1368	317	1367.50	317.26	0.10	0.08	0.18
17	57.30	11.30	7.90	0.600	1433	332	1432.50	332.34	0.10	0.08	0.18
18	58.90	11.30	7.90	0.600	1473	342	1472.50	341.62	0.09	0.08	0.17
19	59.60	11.30	7.90	0.600	1490	346	1490.00	345.68	0.09	0.08	0.17
20	60.30	11.30	7.90	0.151	1508	350	1507.50	349.74	0.03	0.02	0.05
21	62.20	11.30	7.90	0.151	1555	361	1555.00	360.76	0.02	0.02	0.05
22	62.40	11.30	7.90	0.151	1560	362	1560.00	361.92	0.02	0.02	0.05
23	62.70	11.30	7.90	0.151	1568	364	1567.50	363.66	0.02	0.02	0.05
24	63.30	11.30	7.90	0.151	1583	367	1582.50	367.14	0.02	0.02	0.04
**25**	**65.60**	11.30	7.90	0.151	1640	380	1640.00	380.48	0.02	0.02	0.04
26	65.00	11.30	7.90	0.151	1625	377	1625.00	377.00	0.02	0.02	0.04
27	65.70	11.30	7.90	0.151	1643	381	1642.50	381.06	0.02	0.02	0.04
28	65.20	11.30	7.90	0.151	1630	378	1630.00	378.16	0.02	0.02	0.04
29	66.70	11.30	7.90	0.151	1668	387	1667.50	386.86	0.02	0.02	0.04
30	66.30	11.30	7.90	0.151	1658	385	1657.50	384.54	0.02	0.02	0.04
31	67.10	11.30	7.90	0.151	1678	389	1677.50	389.18	0.02	0.02	0.04
32	67.70	11.30	7.90	0.151	1693	393	1692.50	392.66	0.02	0.02	0.04
33	67.00	11.30	7.90	0.151	1675	389	1675.00	388.60	0.02	0.02	0.04
34	66.80	11.30	7.90	0.151	1670	387	1670.00	387.44	0.02	0.02	0.04
35	66.50	11.30	7.90	0.151	1663	386	1662.50	385.70	0.02	0.02	0.04
36	67.20	11.30	7.90	0.151	1680	390	1680.00	389.76	0.02	0.02	0.04
37	66.20	11.30	7.90	0.151	1655	384	1655.00	383.96	0.02	0.02	0.04
38	67.60	11.30	7.90	0.151	1690	392	1690.00	392.08	0.02	0.02	0.04
39	67.80	11.30	7.90	0.151	1695	393	1695.00	393.24	0.02	0.02	0.04
40	69.30	11.30	7.90	0.146	1733	402	1732.50	401.94	0.02	0.02	0.04
41	68.50	11.30	7.90	0.146	1713	397	1712.50	397.30	0.02	0.02	0.04
42	67.60	11.30	7.90	0.146	1690	392	1690.00	392.08	0.02	0.02	0.04
43	67.60	11.30	7.90	0.146	1690	392	1690.00	392.08	0.02	0.02	0.04
44	68.00	11.30	7.90	0.146	1700	394	1700.00	394.40	0.02	0.02	0.04
45	66.60	11.30	7.90	0.146	1665	386	1665.00	386.28	0.02	0.02	0.04

46	68.20	11.30	7.90	0.146	1705	396	1705.00	395.56	0.02	0.02	0.04
47	67.70	11.30	7.90	0.146	1693	393	1692.50	392.66	0.02	0.02	0.04
48	67.50	11.30	7.90	0.146	1688	392	1687.50	391.50	0.02	0.02	0.04
49	67.50	11.30	7.90	0.146	1688	392	1687.50	391.50	0.02	0.02	0.04
50	67.60	11.30	7.90	0.146	1690	392	1690.00	392.08	0.02	0.02	0.04
51	67.90	11.30	7.90	0.146	1698	394	1697.50	393.82	0.02	0.02	0.04
52	68.90	11.30	7.90	0.146	1723	400	1722.50	399.62	0.02	0.02	0.04
53	66.90	11.30	7.90	0.146	1673	388	1672.50	388.02	0.02	0.02	0.04
54	66.20	11.30	7.90	0.146	1655	384	1655.00	383.96	0.02	0.02	0.04
55	67.80	11.30	7.90	0.146	1695	393	1695.00	393.24	0.02	0.02	0.04
56	66.40	11.30	7.90	0.146	1660	385	1660.00	385.12	0.02	0.02	0.04
57	65.80	11.30	7.90	0.146	1645	382	1645.00	381.64	0.02	0.02	0.04
58	65.70	11.30	7.90	0.146	1643	381	1642.50	381.06	0.02	0.02	0.04
59	64.50	11.30	7.90	0.146	1613	374	1612.50	374.10	0.02	0.02	0.04
60	64.80	11.30	7.90	0.185	1620	376	1620.00	375.84	0.03	0.02	0.05
61	64.40	11.30	7.90	0.185	1610	374	1610.00	373.52	0.03	0.02	0.05
62	65.20	11.30	7.90	0.185	1630	378	1630.00	378.16	0.03	0.02	0.05
63	64.60	11.30	7.90	0.185	1615	375	1615.00	374.68	0.03	0.02	0.05
64	61.70	11.30	7.90	0.185	1543	358	1542.50	357.86	0.03	0.03	0.05
65	64.40	11.30	7.90	0.185	1610	374	1610.00	373.52	0.03	0.03	0.06
66	62.90	11.30	7.90	0.185	1573	365	1572.50	364.82	0.03	0.03	0.06
67	64.20	11.30	7.90	0.185	1605	372	1605.00	372.36	0.03	0.02	0.05
68	64.00	11.30	7.90	0.185	1600	371	1600.00	371.20	0.03	0.03	0.05
69	62.70	11.30	7.90	0.185	1568	364	1567.50	363.66	0.03	0.03	0.06
70	59.30	11.30	7.90	0.185	1483	344	1482.50	343.94	0.03	0.03	0.06
71	61.50	11.30	7.90	0.185	1538	357	1537.50	356.70	0.03	0.03	0.06
72	61.70	11.30	7.90	0.185	1543	358	1542.50	357.86	0.03	0.03	0.06
73	59.20	11.30	7.90	0.185	1480	343	1480.00	343.36	0.03	0.03	0.06
74	60.70	11.30	7.90	0.185	1518	352	1517.50	352.06	0.03	0.03	0.06
75	62.30	11.30	7.90	0.185	1558	361	1557.50	361.34	0.03	0.03	0.06
76	58.20	11.30	7.90	0.185	1455	338	1455.00	337.56	0.03	0.03	0.06
77	60.10	11.30	7.90	0.185	1503	349	1502.50	348.58	0.03	0.03	0.06
78	60.70	11.30	7.90	0.185	1518	352	1517.50	352.06	0.03	0.03	0.06
79	58.20	11.30	7.90	0.185	1455	338	1455.00	337.56	0.03	0.03	0.06
80	56.30	11.30	7.90	0.185	1408	327	1407.50	326.54	0.03	0.03	0.06
81	59.10	11.30	7.90	0.185	1478	343	1477.50	342.78	0.04	0.03	0.07
82	59.00	11.30	7.90	0.185	1475	342	1475.00	342.20	0.04	0.03	0.07
83	54.70	11.30	7.90	0.185	1368	317	1367.50	317.26	0.03	0.03	0.06
84	56.60	11.30	7.90	0.185	1415	328	1415.00	328.28	0.03	0.03	0.06
85	55.60	11.30	7.90	0.185	1390	322	1390.00	322.48	0.03	0.03	0.06

Table S4. Determination of THQ and HI in a high exposure scenario

Age (year)	Weight, kg	Contaminant occurrence in milk		Milk consumption	Reference TWI		Calculated		THQ		HI
		Pb (ug/kg)	Cd (ug/kg)	Kg/day	TWI Pb	TWI Cd	TWI Pb	TWI Cd	Pb	Cd	
2	12.40	11.30	7.90	0.600	310	72	310.00	71.92	0.42	0.36	0.78
3	14.40	11.30	7.90	0.600	360	84	360.00	83.52	0.36	0.31	0.67
4	16.10	11.30	7.90	0.600	403	93	402.50	93.38	0.32	0.27	0.59
5	17.90	11.30	7.90	0.600	448	104	447.50	103.82	0.29	0.24	0.53
6	20.10	11.30	7.90	0.720	503	117	502.50	116.58	0.31	0.26	0.56
7	22.00	11.30	7.90	0.720	550	128	550.00	127.60	0.28	0.24	0.51
8	24.60	11.30	7.90	0.720	615	143	615.00	142.68	0.25	0.21	0.46
9	26.80	11.30	7.90	0.720	670	155	670.00	155.44	0.22	0.19	0.41
10	29.60	11.30	7.90	0.720	740	172	740.00	171.68	0.20	0.17	0.37
11	32.70	11.30	7.90	0.720	818	190	817.50	189.66	0.17	0.15	0.32
12	36.40	11.30	7.90	0.720	910	211	910.00	211.12	0.15	0.13	0.28
13	41.90	11.30	7.90	0.720	1048	243	1047.50	243.02	0.14	0.12	0.26
14	47.60	11.30	7.90	0.720	1190	276	1190.00	276.08	0.13	0.11	0.24
15	51.70	11.30	7.90	0.720	1293	300	1292.50	299.86	0.12	0.10	0.22
16	54.70	11.30	7.90	0.720	1368	317	1367.50	317.26	0.12	0.10	0.22
17	57.30	11.30	7.90	0.720	1433	332	1432.50	332.34	0.12	0.10	0.21
18	58.90	11.30	7.90	0.720	1473	342	1472.50	341.62	0.11	0.10	0.21
19	59.60	11.30	7.90	0.720	1490	346	1490.00	345.68	0.11	0.10	0.21
20	60.30	11.30	7.90	0.228	1508	350	1507.50	349.74	0.04	0.03	0.07
21	62.20	11.30	7.90	0.228	1555	361	1555.00	360.76	0.04	0.03	0.07
22	62.40	11.30	7.90	0.228	1560	362	1560.00	361.92	0.04	0.03	0.07
23	62.70	11.30	7.90	0.228	1568	364	1567.50	363.66	0.04	0.03	0.07
24	63.30	11.30	7.90	0.228	1583	367	1582.50	367.14	0.04	0.03	0.07
**25**	**65.60**	11.30	7.90	0.228	1640	380	1640.00	380.48	0.04	0.03	0.07
26	65.00	11.30	7.90	0.228	1625	377	1625.00	377.00	0.04	0.03	0.07
27	65.70	11.30	7.90	0.228	1643	381	1642.50	381.06	0.04	0.03	0.07
28	65.20	11.30	7.90	0.228	1630	378	1630.00	378.16	0.04	0.03	0.07
29	66.70	11.30	7.90	0.228	1668	387	1667.50	386.86	0.03	0.03	0.06
30	66.30	11.30	7.90	0.228	1658	385	1657.50	384.54	0.03	0.03	0.06
31	67.10	11.30	7.90	0.228	1678	389	1677.50	389.18	0.03	0.03	0.06
32	67.70	11.30	7.90	0.228	1693	393	1692.50	392.66	0.04	0.03	0.06
33	67.00	11.30	7.90	0.228	1675	389	1675.00	388.60	0.03	0.03	0.06
34	66.80	11.30	7.90	0.228	1670	387	1670.00	387.44	0.03	0.03	0.06
35	66.50	11.30	7.90	0.228	1663	386	1662.50	385.70	0.03	0.03	0.06
36	67.20	11.30	7.90	0.228	1680	390	1680.00	389.76	0.03	0.03	0.06
37	66.20	11.30	7.90	0.228	1655	384	1655.00	383.96	0.03	0.03	0.06
38	67.60	11.30	7.90	0.228	1690	392	1690.00	392.08	0.03	0.03	0.06
39	67.80	11.30	7.90	0.228	1695	393	1695.00	393.24	0.03	0.03	0.06
40	69.30	11.30	7.90	0.218	1733	402	1732.50	401.94	0.03	0.03	0.06
41	68.50	11.30	7.90	0.218	1713	397	1712.50	397.30	0.03	0.03	0.06
42	67.60	11.30	7.90	0.218	1690	392	1690.00	392.08	0.03	0.03	0.06
43	67.60	11.30	7.90	0.218	1690	392	1690.00	392.08	0.03	0.03	0.06
44	68.00	11.30	7.90	0.218	1700	394	1700.00	394.40	0.03	0.03	0.06
45	66.60	11.30	7.90	0.218	1665	386	1665.00	386.28	0.03	0.03	0.06

46	68.20	11.30	7.90	0.218	1705	396	1705.00	395.56	0.03	0.03	0.06
47	67.70	11.30	7.90	0.218	1693	393	1692.50	392.66	0.03	0.03	0.06
48	67.50	11.30	7.90	0.218	1688	392	1687.50	391.50	0.03	0.03	0.06
49	67.50	11.30	7.90	0.218	1688	392	1687.50	391.50	0.03	0.03	0.06
50	67.60	11.30	7.90	0.218	1690	392	1690.00	392.08	0.03	0.03	0.06
51	67.90	11.30	7.90	0.218	1698	394	1697.50	393.82	0.03	0.03	0.06
52	68.90	11.30	7.90	0.218	1723	400	1722.50	399.62	0.03	0.03	0.06
53	66.90	11.30	7.90	0.218	1673	388	1672.50	388.02	0.03	0.03	0.06
54	66.20	11.30	7.90	0.218	1655	384	1655.00	383.96	0.03	0.03	0.06
55	67.80	11.30	7.90	0.218	1695	393	1695.00	393.24	0.03	0.03	0.06
56	66.40	11.30	7.90	0.218	1660	385	1660.00	385.12	0.03	0.03	0.06
57	65.80	11.30	7.90	0.218	1645	382	1645.00	381.64	0.03	0.03	0.06
58	65.70	11.30	7.90	0.218	1643	381	1642.50	381.06	0.03	0.03	0.06
59	64.50	11.30	7.90	0.218	1613	374	1612.50	374.10	0.03	0.03	0.06
60	64.80	11.30	7.90	0.278	1620	376	1620.00	375.84	0.04	0.04	0.08
61	64.40	11.30	7.90	0.278	1610	374	1610.00	373.52	0.04	0.04	0.08
62	65.20	11.30	7.90	0.278	1630	378	1630.00	378.16	0.04	0.04	0.08
63	64.60	11.30	7.90	0.278	1615	375	1615.00	374.68	0.04	0.04	0.08
64	61.70	11.30	7.90	0.278	1543	358	1542.50	357.86	0.04	0.04	0.08
65	64.40	11.30	7.90	0.278	1610	374	1610.00	373.52	0.05	0.04	0.08
66	62.90	11.30	7.90	0.278	1573	365	1572.50	364.82	0.05	0.04	0.08
67	64.20	11.30	7.90	0.278	1605	372	1605.00	372.36	0.04	0.04	0.08
68	64.00	11.30	7.90	0.278	1600	371	1600.00	371.20	0.04	0.04	0.08
69	62.70	11.30	7.90	0.278	1568	364	1567.50	363.66	0.05	0.04	0.08
70	59.30	11.30	7.90	0.278	1483	344	1482.50	343.94	0.05	0.04	0.09
71	61.50	11.30	7.90	0.278	1538	357	1537.50	356.70	0.05	0.04	0.09
72	61.70	11.30	7.90	0.278	1543	358	1542.50	357.86	0.05	0.04	0.09
73	59.20	11.30	7.90	0.278	1480	343	1480.00	343.36	0.05	0.04	0.09
74	60.70	11.30	7.90	0.278	1518	352	1517.50	352.06	0.05	0.04	0.09
75	62.30	11.30	7.90	0.278	1558	361	1557.50	361.34	0.05	0.04	0.09
76	58.20	11.30	7.90	0.278	1455	338	1455.00	337.56	0.05	0.04	0.09
77	60.10	11.30	7.90	0.278	1503	349	1502.50	348.58	0.05	0.04	0.09
78	60.70	11.30	7.90	0.278	1518	352	1517.50	352.06	0.05	0.04	0.09
79	58.20	11.30	7.90	0.278	1455	338	1455.00	337.56	0.05	0.04	0.09
80	56.30	11.30	7.90	0.278	1408	327	1407.50	326.54	0.05	0.04	0.09
81	59.10	11.30	7.90	0.278	1478	343	1477.50	342.78	0.05	0.05	0.10
82	59.00	11.30	7.90	0.278	1475	342	1475.00	342.20	0.05	0.05	0.10
83	54.70	11.30	7.90	0.278	1368	317	1367.50	317.26	0.05	0.04	0.09
84	56.60	11.30	7.90	0.278	1415	328	1415.00	328.28	0.05	0.04	0.10
85	55.60	11.30	7.90	0.278	1390	322	1390.00	322.48	0.05	0.04	0.09
